# Spectrofluorimetric Protocol for Estimation of Commonly Used Antispasmodic Drotaverine. Fluorescence Quenching Study

**DOI:** 10.1007/s10895-023-03487-7

**Published:** 2023-11-06

**Authors:** Sayed M. Derayea, Nihad A. Mahmoud, Deena A. M. Noureldeen, Tamer Z. Attia

**Affiliations:** Analytical Chemistry Department, Faculty of Pharmacy, Minia University, Minia, Egypt

**Keywords:** Phosphodiesterase- IV inhibitor, Eosin Y, Ion- pair complex, Drotaverine, Antispasmodics

## Abstract

A fast, simple, accurate, precise, and cheap fluorimetric protocol has been proposed for analysis of a phosphodiesterase-IV inhibitor, namely drotaverine hydrochloride. Fluorimetric protocol is based on estimating the decrease in the eosin Y fluorescence intensity by quantitative addition of drotaverine at pH 3.1 (acetate buffer). An ion pair complex is formed, which leads to quenching in the fluorescence intensity of the dye without need of prior extraction at 534 nm (λ_ex_. 339 nm). Different reaction perimeters which influence the production of complex (ion pair between drotaverine and eosin) were deeply investigated and optimized. The developed fluorimetric protocol is capable for quantitative estimation of drotaverine in linear range of 0.4 to 2.5 µg mL^−1^. After method validation in respect to ICH guidelines, it was applied to determine drotaverine in its commercial preparation. By comparing with other reported method, the developed and validated fluorimetric protocol is capable for estimation of drotaverine in commercial preparation with good accuracy and excellent precision.

## Introduction

Gastrointestinal drugs are used to treat gastrointestinal disorders and include many different classes, such as: antacids, antidiarrheal, digestive enzymes, 5 amino salicylates and functional bowel disorder drugs. One of these categories is antispasmodics, which can relieve spasm by attaching to the muscarinic receptors, leading to preventing the chemicals from attaching there so the muscle cannot contract, leading to relieving the symptoms caused by irritable bowel syndrome. One of the most commonly used antispasmodics is drotaverine [1].

Drotaverine, a selective phosphodiesterase inhibitor, is widely used in the treatment of various smooth muscle spasm-related disorders, including gastrointestinal, biliary, and genitourinary tract disorders [2]. In addition, drotaverine is therapeutically applied during labor to enhance cervical dilation. Furthermore, it could be considered as pain relief drug applied for treatment of gastrointestinal pain, biliary and kidney stone pain, and abdominal pain [3]. Therefore, the accurate determination of drotaverine is of great importance for pharmaceutical analysis and clinical research purposes. The chemical structure of the studied drug is presented in Table 1.

**Table 1:** Chemical structure, log_P_ and Pk_a_ of drotaverine

**Name**	**Chemical structure**	**log**_**p**_	**Pk**_**a**_
Drotaverine		4.19	7.11

As the great clinically significant and wide use of drotaverine, numerous analytical techniques have been published for its quantitative estimation in various matrices, including spectrophotometry [4–12], spectrofluorimetry [13, 14], high performance liquid chromatography [4, 15–19], thin layer chromatography [4, 20], liquid chromatography MS/MS [21], membrane selective electrode [22], potentiometry [23, 24], and voltammetry [25].

Spectrofluorimetry has emerged as a sensitive and reliable analytical technique for the determination of pharmaceutical compounds due to its high selectivity and sensitivity. Spectrofluorimetric methods offer advantages such as simplicity, rapidity, and cost-effectiveness, making them suitable for routine analysis in pharmaceutical laboratories.

On the other hand, other reported techniques for drotaverine analysis require well trained person or highly sophisticated instrument such in the case of chromatographic or electrochemical methods. In addition, sensitivity of spectrofluorimetric technique is superior to that of the spectrophotometric methods.

Although, two spectrofluorimetric methods were published for the analysis of drotaverine, these methods suffered from several limitations including the use of hazardous solvent as sulphuric acid which require a great caution in its use [13] or utilizing of expensive reagent [14]. Therefore, the establishment of a cost effective, highly sensitive and reliable spectrofluorimetric method for drotaverine determination in its pure form and commercial tablet without prior extraction is of great importance.

Generally, drugs with cationic basic nitrogen group react via ion pair complexation with anionic acidic tetrabromofluorescein (Eosin, green fluorescence) in acidic medium, resulting in quenching of the dye native fluorescence [26, 27]. The objective of the present article is to develop and validate a spectrofluorimetric method for the quantitative estimation of drotaverine in authentic and pharmaceutical preparation with higher sensitivity and cost effectiveness. The protocol of the proposed method is depending simply on binary complex formation between drotaverine and tetrabromofluoresce in a buffer aqueous solution. The method's specificity, linearity, precision, accuracy, and robustness will be extensively evaluated using validation parameters recommended by ICH guiding rules.

## Experimental

### Apparatus

Luminescence spectrometer established with a 150-W xenon lamp (Perkin Elmer LS; United Kingdom) was used for carrying out fluorescence estimation. The measurements were carried out by using quartz cell (1 cm bath length). Two monochromators were used one for excitation and the other for emission with 10 nm as a slit width. FL WinLab software connected to luminescence spectrometer was utilized for data collection. A SONICOR Sonicator (SC-101TH) and ADIIP PH-meter (Adwa, Romania) were utilized throughout investigation. Weighing was performed on digital analytical balance (Mettler Toledo, Glattbrugg, Switzerland).

### Materials and Reagents

Drotaverine authentic sample (99% purity) was afforded from Sunny Pharmaceutical Co., SAE Industrial Zone (Badr City, Cairo, Egypt). In 100 mL volumetric flask, disodium salt of 0.971 × 10^–4^ M eosin (Merck, Darmstadt, Germany) was composed by solvating 24 mg eosin in distilled water followed by dilution of 7 mL eosin to 25 mL with same solvent. 0.2 M solution of acetate buffer (pH 3.1) was composed by combining proper volumes of 0.2 M acetic acid and sodium acetate.

### Pharmaceutical Formulations

Spasmocure^®^ ampoules (40 mg/2 mL; Sunny Pharmaceutical Co., SAE Industrial Zone, Badr City, Cairo, Egypt) are identified to carry 40 mg of drotaverine hydrochloride in one ampoule.

### Standard Drug Solutions

In volumetric flask (100 mL), drotaverine stock solution (100 µg mL^−1^) was composed by solvating 10.0 mg in distilled water. Then working solution of drotaverine (20 µg mL^−1^) was used by adding water to 5 ml of stock solution in 25 mL volumetric flask. Standard stock solution was stable for at least seven days when refrigerated.

### Experimental Procedure for Drotaverine Quantitative Analysis

Different volumes of working standard solution of drotaverine were transferred into a 10 mL calibrated flask, followed by addition of 1.3 ml acetate buffer (PH 3.1) and 2 mL eosin (9.71 × 10^–2^ mM) solutions. Distilled water was added to the solution mixture up to the mark. The mixture was excited at 339 nm and fluorescence intensity difference of the resultant product emission against eosin was measured at 534 nm. Controlled experimental procedure was carried out at the same time in the same manner, except addition of drotaverine.

### Analysis of Spasmocure^®^ Ampoules

In volumetric flask, 0.1 mL of the ampoule content was taken (equivalent to 2000 µg of drotaverine) and water was added up to the mark to form 20 µg mL^−1^ drotaverine solutions. Different volumes of drotaverine solution were taken and estimated as described before.

## Results and Discussion

Although spectrofluorimetic method of analysis has been considered the technique of choice for analysis of several pharmaceutical drugs, only two fluorimetric methods have been published for quantitative analysis of drotaverine. In our study, a spectrofluorimetric protocol has been developed based on ion pair complexation of drotaverine with eosin. The quenching degree in eosin fluorescence is directly proportional to the concentration of drotaverine added which could be used for quantitative measure of drotaverine in its pure form and its ampoules. The proposed protocol has several advantages over the previously reported fluorimetic methods [13, 14]. The previously reported fluorimetric method [13] is based on quantitative estimation of drotaverine by measuring of drotaverine fluorescence intensity in sulphuric acid medium. However, our proposed method is based on measurement of eosin fluorescence quenching in presence of acetate buffer using distilled water as diluting solvent. Therefore, the proposed protocol could be considered greener than the reported method [13] by avoiding use of hazard solvent. Furthermore, the proposed protocol is based on use more cheap and available reagents than the previously reported fluorimetric method [14].

### Fluorescence Spectra

**Fig. 1:**
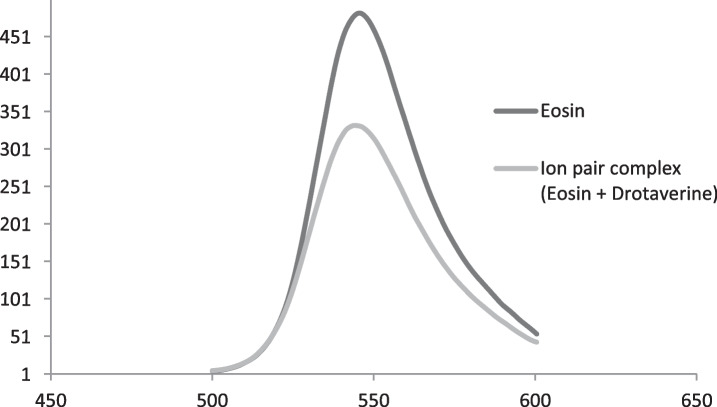
Emission spectrum of eosin only and eosin after addition of 2 µg mL^−1^ drotaverine in presence of acetate buffer

At 534 nm, the eosin emission intensity was detected upon excitation at 339 nm. The formation of eosin-drotaverine complex (ion pair) resulted in a significant decrease in fluorescence after drotaverine addition to eosin-Y (Fig. 1). The decline in eosin fluorescence was traced after each drotaverine addition. Linear relationship could be observed between the eosin fluorescence quenching (∆F) and the added drotaverine concentrations.

### Experimental Conditions Optimization

Several parameters had been studied to investigate the optimal experimental conditions for the formed complex. These specifications were solution pH, reaction time and eosin volumes.

#### Buffer pH and Volume

**Fig. 2:**
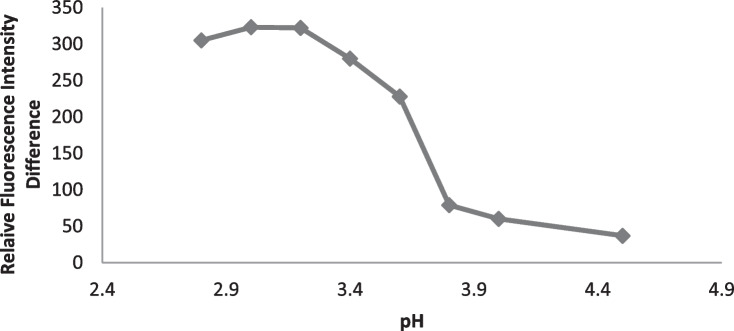
Effect of pH on the reaction of the studied drug (2.5 µg mL^−1^) of Drotaverine

**Fig. 3:**
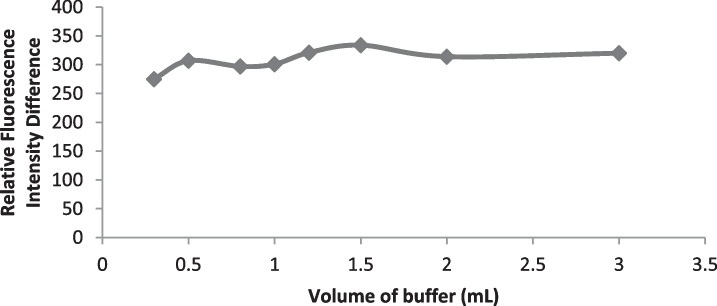
Effect of acetate buffer volume on the reaction of the studied drug (2.5 μg mL^−1^) of Drotaverine

The effect of pH on the fluorescence quenching value (∆ F) had been carefully investigated at pH range of 2.8—4.5 (Fig. 2) by using acetate buffer. The fluorescence difference increase up to pH 3 then it was stable to pH 3.2. At PH higher than 3.2, there was significant decrease in the fluorescence difference. Therefore, the optimum pH selected for further investigation was 3.1 Furthermore; the effect of acetate buffer volumes (0.3—3.0 mL) was examined (Fig. 3). When the volume of the buffer solution increased, a slightly raise in the fluorescence difference was observed as far as reaching 1.2 mL and no change occurred up to 1.5 mL. Therefore, 1.3 mL of acetate buffer solution at pH 3.1 was selected in the experimental procedures of drotaverine quantitation.

#### Eosin Volume

**Fig. 4:**
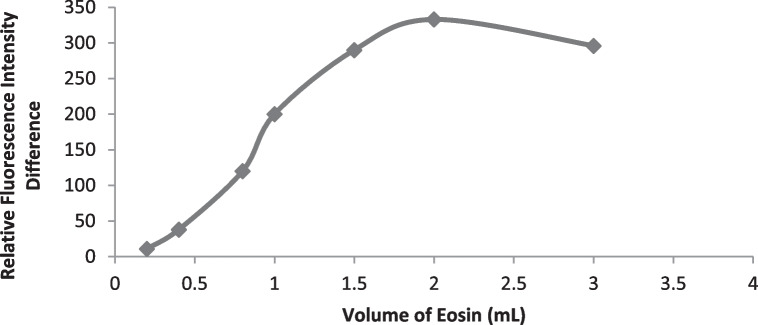
Effect of volume of Eosin on the reaction of the studied drug (2.5 μg mL^−1^) of Drotaverine

By adding different volumes of eosin (0.2 -3.0 mL) to drotaverine, there was a significant increase in fluorescence intensity difference. The maximum fluorescence quenching value was achieved by using 1.5 ml of eosin (Fig. 4). Adding more eosin resulted in no marked changes in fluorescence quenching values up to 3.0 ml. As a result, 2.0 ml of eosin solution (9.7 × 10^–2^ mM) was used throughout the investigation.

#### Time Effect and Complex Stability

**Fig. 5:**
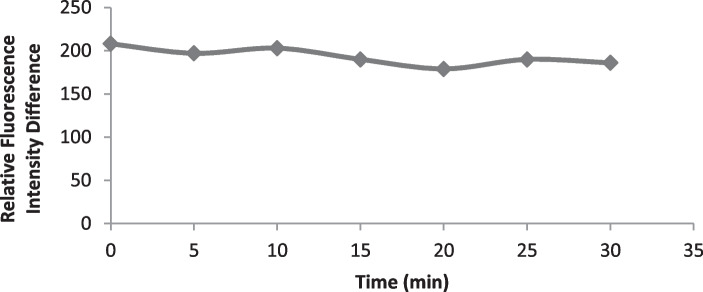
Effect of time on the reaction of the studied drug (1.6 μg mL^−1^) of Drotaverine

At ambient temperature and just after combination of drotaverine and the dye solution, the reaction was completed. Moreover, the originated complex was stable for at least 30 min after the final dilution (Fig. 5) as the fluorescence quenching effect did not change over this time. Accordingly, the fluorescence emission measurements were examined just after combining the drug and eosin without standing.

### Method's Validation

Several parameters had been studied including linearity, limits of detection and quantification, accuracy, precision, and robustness to determine quality and validity of proposed fluorimetric protocol confirming to ICH Q2 guidelines [28].

#### Linearity and Range

By using the previously discussed optimized parameters for carrying out the fluorimetric assay, calibration curve was fabricated by using different concentrations of drotaverine. Calibration curve was made by tracing the decline in eosin's fluorescence values on y-axis upon mixing with serial drotaverine's concentrations on x-axis. Relationship was found to be linear between the fluorescence difference and drotaverine concentrations as indicated by high correlation coefficient value of about 0.9997. Slope with its standard deviation and intercept with its standard deviation were computed and summarized in Table 2. Declining of eosin fluorescence upon adding drotaverine was in a linear over the drotaverine's concentration range from 0.4 µg mL^−1^ up to 2.5 µg mL^−1^.

**Table 2:** Summary of quantitative parameters and statistical data of the proposed method

**Parameter**	**Result**
Linear Range^*^ (µg mL^**−1**^**)**	0.4–2.5
**Slope**	115.5
**S.D (standard deviation) of slope**	1.309
**Intercept**	34.394
**S.D (standard deviation) of intercept**	2.042
**R (correlation coefficient)**	0.9997
**R**^**2** ^**(determination coefficient)**	0.9995
**LOD (limit of detection)**	0.058 µg mL^−1^
**LOQ (limit of quantification)**	0.177 µg mL^−1^

*Number of determinations for construction of calibration curve is eight

#### Detection and quantitation limits

To figure out the detection (LOD) and quantitation (LOQ) limits, the following formulations; LOD = 3.3б /S and LOQ = 10б /S (б; the intercept's standard deviation & S; the slope) have been used. The estimated limits values were found to be of 0.058 and 0.177 µg mL^−1^ for LOD and LOQ, respectively. These numerical data point to extraordinary sensitivity of the fluorimetric assay protocol.

#### Accuracy

The accuracy of the proposed fluorimetric protocol was verified by using three different concentrations 0.4, 1.0, and 1.6 µg mL^−1^. The results were summarized in Table 3 and represented by percentage recovery and relative standard deviation. The percentage recovery values were in the range from 99.60% to 101.13%, which means good accuracy of the developed method and its suitability for quality control measurements.

**Table 3:** Evaluation of the accuracy of the proposed spectrofluorimetric method

**Sample no.**	**Taken conc.** **(µg mL** ^**−1**^**)**	Found conc. * (µg mL ^**−1**^**)**	**Recovery %**
**1**	0.4	0.398	99.60
**2**	1.0	1.011	101.13
**3**	1.6	1.61	100.67
**Mean**			100.47
**SD**			0.785
**RSD**^#^			0.781

^#^RSD relative standard deviation; *mean of three replicate measurements

#### Precision

Three different concentration's levels (0.4, 1.0, and 1.6 µg mL^−1^) of the working standard drug solutions for checking the intra-day precision of the developed method were investigated on the day. Inter-day precision was evaluated by replicating fluorimetric assay on three distinguishable days. Data of evaluated different precision levels are tabulated in Table 4. The estimated relative standard deviation numerical values were less than 2%, meaning acceptable reproducibility and repeatability of the fluorimetric assay protocol.

**Table 4:** Intra- and inter-day precisions for the analysis of drotaverine by the proposed spectrofluorimetric method

**Concentration level (µg mL**^**−1**^**)**	%Recovery* ± SD		**RSD**^#^
	**Intra-day**	**Inter-day**	
**0.4**	99.93 ± 1.53	100.30 ± 1.24	1.53
**1.0**	99.78 ± 0.79	100.07 ± 1.30	0.79
**1.6**	101.86 ± 0.31	101.29 ± 0.59	0.30

*Mean of three determinations. ^#^Relative standard deviation for intra-day precisiion evaluation

#### Robustness

Robustness of the proposed fluorimetric protocol has been checked to clarify the influence of introducing minor changes in the experimental conditions, such as buffer volume, buffer pH, eosin volume, and reaction time, on the accuracy of measurements. From the result in Table 5, it was concluded that minor changes in experimental parameters have no effect on accuracy of the measurements as % recoveries were nearly in the range of 98 – 102%.

**Table 5:** Robustness of the proposed method for determination of drotaverine

**Parameters**	**Minor changes in experimental parameters**		
Buffer pH	3.0	3.1	3.2
% recovery	99.95	98.91	99.60
Buffer Volume	1.2 mL	1.3 mL	1.5 mL
% recovery	99.26	99.95	101.68
Eosin Volume	1.8 mL	2.0 mL	2.2 mL
% recovery	100.29	102.02	100.64
Time	0 min	5 min	10 min
% recovery	100.20	98.31	101.93

### Application to Spasmocure^®^ Ampoules

Fluorimetric assay protocol was experimentally used to determine the drug contents of Spasmocure^®^ ampoules. The estimated data were compared statistically with reported method's data [11] in regard to precision and accuracy (Table 6). The proposed spectrofluorietric protocol and other reported method have been applied for determination of five different concentration of drotaverine within the linearity range in both methods. Each concentration was repeated for three times and the results were expressed as percentage recoveries and standard deviation. The results obtained for analysis of drotaverine by two methods were compared by student's *t*-test. It was observed that calculated *t-*value (1.87) was less than tabulated one (2.306) providing evidence for the acceptable accuracy of the method. Furthermore, the obtained data were also compared by F-test. The calculated *F*-value (2.14) was less than tabulated one (6.338). There were no marked differences between the two methods' results whereas the tabulated values for *t-* and *F-*test were higher than the calculated ones. These verify the applicability of the proposed method for assay of drotaverine in its dosage form by high degree of accuracy and precision.

**Table 6:** Statistical analysis of the obtained results using the proposed spectrofluorimetric and reported method for analysis of drotaverine in commercial preparation

**Investigated** **Drugs**	**Commercial** **Preparation**	**Proposed Method** ** ± SD (n = 5)**	**Reported Method** ** ± SD (n** **= 5)**
**Drotaverine**	Spasmocure^®^ ampoule	100.03 ± 1.30	101.95 ± 1.90
		t* = 1.87, F* = 2.14	

*Tabulated values at 95% confidence limit*; t* = 2.306 and* F* = 6.338

## Conclusion

A green, economic, instantaneous, and sensitive fluorescence quenching protocol was adapted for drotaverine analysis. The protocol is contingent on ion-pair complexation with eosin which has low-price and available in the market. Due to the protocol's high sensitivity, it could estimate drotaverine at very low concentration of approximately 0.177 µg mL^−1^. Contrasted with HPLC analytical techniques, fluorimetric techniques of analysis are relatively easy, less laborious and quick. Due to using of water as solvent with no need for any polluting or organic solvent for extraction or dilution, the proposed fluorimetric protocol satisfies the preliminary conditions of green chemistry. As such, the proposed fluorimetric protocol could be fruitful for assay of drotaverine in quality control purposes.

## Data Availability

Not applicable.

## References

[1] Rai RR, Dwivedi M, Kumar N (2014) Efficacy and safety of drotaverine hydrochloride in irritable bowel syndrome: A randomized double-blind placebo-controlled study. Saudi J Gastroenterol 20(6):378–382 10.4103/1319-3767.145331 PMC4271014 25434320

[2] The Drug Bank Database: Drotaverine". Drug Bank Version 4.1. The Metabolomics Innovation Centre. Retrieved 9 August 2014

[3] Singh KC, Jain P, Goel N, Saxena A (2004) Drotaverine hydrochloride for augmentation of labor. Int J Gynaecol Obstet 84(1):17–22 14698825 10.1016/s0020-7292(03)00276-5

[4] Metwally FH, Abdelkawy M, Naguib IA (2006) Determination of nifuroxazide and drotaverine hydrochloride in pharmaceutical preparations by three independent analytical methods. J AOAC Int 89(1):78–87 16512232

[5] Hedjazi M, Vishnikin AB, Balanenko AD (2021) A Green Spectrophotometric Method for Determination of Drotaverine Hydrochloride in Pharmaceutical Preparations using Formation of Ion Association Complex with Erythrosine. J Chem Technol 29(3):467–475

[6] Anumolu PD, Gurrala S, Yeradesi VR, Puvvadi SBR, Chaval SVS (2013) Development of Dissolution Test Method for Drotaverine Hydrochloride/Mefenamic Acid Combination Using Derivative Spectrophotometry. Trop J Pharm Res 12(2):227–232

[7] Patel RK, Patel RN, Ganure AL, Patel LJ (2012) Simultaneous Estimation of Ranitidine and Drotaverine in Combined Pharmaceutical Dosage Form by Derivative Spectrophotometric Method. Asian J Research Chem 5(02):215–217

[8] Das V, Sambherao A, Gajare S, Zalte A, Saudagar RB (2016) UV-Spectroscopic estimation and validation of drotaverine concentration in bulk and dosage form. Asian J Pharm Anal 6(3):188–190

[9] Abdallah OM, Rashed NS, El-Olemy A, Hosam Eldin AI (2017) UV Spectrophotometric determination of paracetamol in presence of drotaverine hydrochloride. J Adv Pharmacy Res 1(2):89–95

[10] Amin AS, El-Sheikh R, Zahran F, Gouda AA (2007) Spectrophotometric determination of pipazethate HCl, dextromethorphan HBr and drotaverine HCl in their pharmaceutical preparations. Spectrochim Acta A Mol Biomol Spectrosc 67(3–4):1088–1093 10.1016/j.saa.2006.09.027 17092767

[11] Dahivelkar PP, Bari SB, Shirkhedkar AA (2007) UV Spectrophotometric determination of drotaverin hydrochloride in bulk and pharmaceutical formulation. Asian J Chem 19(4):3245–3246

[12] Rele R (2018) UV-Spectrophotometric Estimation of Drotaverine Hydrochloride by Derivative Method in Pharmaceutical Dosage Form. Int J ChemTech Res 11(10):353–360

[13] El-Wasseef DR, El-Sherbiny D, Eid M, Belal F (2008) Spectrofluorometric determination of drotaverine hydrochloride in pharmaceutical preparations. Anal Lett 41(13):2354–2362

[14] Yegorova A, Leonenko I, Aleksandrova D, Scrypynets Y, Aleksandrova A (2013) Determination of drotaverine hydrochloride in dosage forms by its quenching effect on the luminescence of terbium complex. Journal of Applied Pharmaceutical Science 3(05):006–011

[15] Iqbal MS, Shah PA, Khan T, Syed HK, Qamer N, Pervaiz A, Zaidi HA, Khan SUD (2021) Rapid analytical method development and validation of combined tablet of drotaverine hydrochloride and piroxicam. Asian J Pharm 15(1):100–105

[16] Issa YM, Hassouna ME, Zayed AG (2012) Simultaneous determination of paracetamol, caffeine, domperidone, ergotamine tartrate, propyphenazone, and drotaverine HCl by high performance liquid chromatography. J Liq Chromatogr Relat Technol 35(15):2148–2161

[17] Wahab SU, Mohamed SU, Firthouse S, Mohamed Halith, MC, Sirajudeen SK (2011) Development of rp-hplc method for the simultaneous determination of mefenamic acid and drotaverine hcl combined tablet dosage form. Int J Pharm Sci 3(2):115–117

[18] Hassouna ME, Issa YM, Zayed AG (2014) Determination of residues of acetaminophen, caffeine, and drotaverine hydrochloride on swabs collected from pharmaceutical manufacturing equipment using hplc in support of cleaning validation. J AOAC Int 97(5):1439–1445 10.5740/jaoacint.12-475 25902997

[19] Belal FF, Sharaf El-Din MK, Tolba MM, Elmansi H (2015) Determination of two ternary mixtures for migraine treatment using HPLC method with ultra violet detection. Sep Sci Technol 50(4):592–603

[20] Metwally FH, El-Saharty YS, Refaat M, El-Khateeb SZ (2007) Application of Derivative, Derivative Ratio, and Multivariate Spectral Analysis and Thin-Layer Chomatography-Densitometry for Determination of a Ternary Mixture Containing Drotaverine Hydrochloride, Caffeine, and Paracetamol. J AOAC Int 90(2):391–404 17474510

[21] Vancea S, Gáll Z, Donáth-Nagy G, Borka-Balás R (2014) Rapid LC–MS/MS method for determination of drotaverine in a bioequivalence study. J Pharm Biomed Anal 98:417–423 25005892 10.1016/j.jpba.2014.06.029

[22] El-Saharty YS, Metwaly FH, Refaat M, El-Khateeb SZ (2006) Application of new membrane selective electrodes for the determination of drotaverine hydrochloride in tablets and plasma. J Pharm Biomed Anal 41(3):720–724 16469468 10.1016/j.jpba.2005.11.045

[23] Issa YM, Ibrahim H, Abu-Shawish HM (2005) Carbon Paste Electrode for the Potentiometric Flow Injection Analysis of Drotaverine Hydrochloride in Serum and Urine. Microchim Acta 150:47–54

[24] Ibrahim H, Issa YM, Abu-Shawish HM (2005) Potentiometric Flow Injection Analysis of Drotaverine Hydrochloride in Pharmaceutical Preparations. Anal Lett 38(1):111–132 10.1016/j.jpba.2004.08.032 15620532

[25] Zayed SIM, Issa YM (2009) Cathodic adsorptive stripping voltammetry of drotaverine hydrochloride and its determination in tablets and human urine by differential pulse voltammetry. Bioelectrochemistry 75(1):9–12 19138886 10.1016/j.bioelechem.2008.11.008

[26] Sayed M (2014) Derayea, An application of eosin Y for the selective spectrophotometric and spectrofluorimetric determination of mebeverine hydrochloride. Anal Methods 6:2270–2275

[27] Walash M, Belal F, El-Enany N (2010) ElmansiH, Spectrophotometric and spectrofluorimetric methods for the determination of dothiepin hydrochloride in its pure and dosage forms using eosin. Int J Biomed Sci 6:327–334 23675210 PMC3615291

[28] ICH I (2005) Q2 (R1): Validation of analytical procedures: text and methodology. In International Conference on Harmonization, Geneva

